# Autistic traits shape neuronal oscillations during emotion perception under attentional load modulation

**DOI:** 10.1038/s41598-023-35013-x

**Published:** 2023-05-20

**Authors:** Mathilde Marie Duville, David I. Ibarra-Zarate, Luz María Alonso-Valerdi

**Affiliations:** Tecnologico de Monterrey, Escuela de Ingeniería y Ciencias, Ave. Eugenio Garza Sada 2501, 64849 Monterrey, NL México

**Keywords:** Human behaviour, Attention, Perception, Cognitive neuroscience, Emotion, Social neuroscience

## Abstract

Emotional content is particularly salient, but situational factors such as cognitive load may disturb the attentional prioritization towards affective stimuli and interfere with their processing. In this study, 31 autistic and 31 typically developed children volunteered to assess their perception of affective prosodies via event-related spectral perturbations of neuronal oscillations recorded by electroencephalography under attentional load modulations induced by Multiple Object Tracking or neutral images. Although intermediate load optimized emotion processing by typically developed children, load and emotion did not interplay in children with autism. Results also outlined impaired emotional integration emphasized in theta, alpha and beta oscillations at early and late stages, and lower attentional ability indexed by the tracking capacity. Furthermore, both tracking capacity and neuronal patterns of emotion perception during task were predicted by daily-life autistic behaviors. These findings highlight that intermediate load may encourage emotion processing in typically developed children. However, autism aligns with impaired affective processing and selective attention, both insensitive to load modulations. Results were discussed within a Bayesian perspective that suggests atypical updating in precision between sensations and hidden states, towards poor contextual evaluations. For the first time, implicit emotion perception assessed by neuronal markers was integrated with environmental demands to characterize autism.

## Introduction

Emotional content is processed as salient sensory information, triggering attentional prioritization supported by bottom-up allocation that may be partially independent from voluntary top-down processes. Indeed, even when emotional processing is not explicitly required, affective information cannot be fully ignored and interferes with cognitive performance^1^.

Cognitive processes involved in emotional integration can be assessed by extracting Event-Related brain Spectral Perturbations (ERSP) that provide indexes of modulations for ongoing neuronal activity, induced by the processing of stimuli. Such activity is highlighted by time–frequency analysis (i.e., increase or decrease of power over time across the frequency band of interest when the subject processes a particular stimulus, independently of the phase of the waveform). Thus, ERSP provide insights into the dynamic shifts of excitation and inhibition in neuronal networks associated with cognitive processes. The synchronization of neuronal oscillations contributes to an increase in time–frequency power at the field potential level, compared to a baseline time-window chosen to be before the occurrence of the event. Thus, increase in oscillatory power is referred to as Event-Related Synchronization (ERS) while decrease reflects Desynchronization (ERD).

Alpha (~ 8–12 Hz) ERS portrays rhythmic cortical inhibition of task-irrelevant brain regions that leads to synchronous firing of a neuronal population. On the other hand, smaller amplitudes, and ERD are associated with a release of cortical inhibition that favors neuronal excitability, active information integration, and cognitive load processes. Alpha ERD have been observed after processing emotional pictures^2^, music, speech, vocalizations^3^, and written linguistic information^4^, outlining a particular engagement of attention, and enhanced perceptual analysis for arousing stimuli compared to neutral ones. Adjacent-to-alpha, low-beta (~ 13–20 Hz) desynchronization also occurs after integrating emotions conveyed by pictures^5^ and affective videos^6^, as an index of motor systems involved in empathy processes. Such “embodied emotion” is sustained by decreases in the power of alpha and beta oscillations (mu rhythm). On the other hand, high-beta oscillations (~ 21–30 Hz) have proved to be increased after emotion processing as an index of maintenance of the current cognitive state^7^. Theta (~ 4–7 Hz) and gamma (> 30 Hz) rhythms ERS have been observed to be triggered by emotional perception. Neuronal synchronization in theta oscillations is involved in memory encoding and retrieval of motivational significance and is outlined during affective content processing^8^, which may reflect the relevance of emotional over neutral information. Finally, higher gamma amplitudes denote cognitive efforts for sensorimotor integration, attention allocation, and memory processes, a pattern that has been observed during emotion perception^2,7^.

Emotion perception is a multi-step mechanism with a time sequence revealing progressive neuronal representations from perceptual detection to integration and evaluation. First stage sensorial recognition occurs during the first 100 ms after stimulus apparition^9^. After low-level acoustic feature extraction, categorization of auditory objects, and emotional salience are integrated approximately 200 ms after stimulus occurrence^10^. Finally, affective motivational relevance is encoded by higher-order cognitive processes between 400 and 1000 ms after stimulus^9,11^.

Autism Spectrum Disorders (ASD) are characterized by impairments in the perception and integration of emotional prosody. Findings from functional magnetic resonance outlined the activation of a wider-spread network that was associated with poorer emotion recognition and higher attentional and cognitive loads for autistics to integrate emotional prosodies^12,13^. The analysis of event-related potentials from electroencephalographic (EEG) data revealed reduced Mismatch Negativity in ASD, a ~ 150–250 ms component that reflects early detection when a relevant acoustic change has been detected. Subsequent involuntary attentional reallocation, associated with the P3a (~ 300–450 ms) has also evidenced weaker and impaired emotional discrimination^14^. Finally, later-stage potentials (~ 400–1000 ms) revealed lower contextual evaluation, interpretation, and memory representations of affective information associated with more severe autistic traits^15^. Spectral analysis on EEG data collected on fathers of ASD children while viewing anger, happiness, and sadness emotional faces revealed disturbed theta and beta patterns over occipital, parietal and central cortices^16^. ERSP analysis before and after neurofeedback training meant to improve social interactions and emotional responsiveness in autistic children showed enhanced alpha mu suppression at early and late stages of emotional faces processing that coincided with better emotion recognition and better social responsiveness^17^. Finally, heightened cognitive efforts were evidenced by increased early and late gamma ERS observed over frontal, central, parietal and occipital sensors after autistic children viewed emotional faces^18^.

A relevant factor in determining the impact of emotional stimuli on behavioral and neuronal perceptions is whether affective information acts as target (task-relevant) or distractor (task-irrelevant). In emotion-irrelevant scenarios, top-down executive control systems attempt to mobilize attentional resources towards target information, but particularly salient affective stimuli compete for reallocation^19^. The Load theory suggests that under higher cognitive demand induced by task-relevant content, attentional control resources are depleted^20^. In such cases, task-irrelevant content is less efficiently suppressed which results in increased processing of irrelevant distractors^21^. However, contradictory results showed that in scenarios where affective stimuli (task-irrelevant) were presented concomitantly with a cognitive task, the ability to understand and make inference about other people’s emotional state was reduced with increased cognitive demand from task-relevant information processing^22^, suggesting reduced emotional awareness being triggered by cognitive load.

Autistic perceptual mechanisms are shaped by local and fine-grained information processing induced by higher reliance on sensorial accuracy. Experimental findings have led to existing theories suggesting reduced top-down control resulting in lower ability for global processing (Weak-Central Coherence, WCC)^23^, or enhanced bottom-up functioning triggering sensorial noise reduction (Enhanced Perceptual Functioning, EPF)^24^. In this sense, both under-precise generalization capacity and heightened sensorial precision would lead to “overfitting” attentional systems to sensorial information and would result in perceptions less sensitive to context^25^. Although both theories may find empirical bedrocks, the overall evidence fails to support any of the proposed frameworks for the autistic perception^26^. Rather, a Bayesian perspective may more accurately describe its dynamics by proposing that unbalanced precision-weighting between sensory processing and prior beliefs may lead to inferences closer to sensorial inputs, and less sensitive to abstract and contextual representations. Predictions are constantly generated about future sensorial inputs based on incoming stimuli and associated prior knowledge, to be compared to current inputs. Whenever the prediction error is higher than the expected variability of sensory inputs, the priors must be updated to solve future discordances. Otherwise, the precision (i.e., confidence) to the prediction error may be lowered. Autistic perception may be shaped by overfitting priors to noisy information through unusual high precision attributed to prediction errors^27^, or to sensory information^28^, consistent with the EPF. Nevertheless, as initially proposed, perception may also be closer to the input if priors are broader (weakened) leading to fewer constraints and higher uncertainty^29^, consistent with the WCC. Accordingly, autistics may attribute an unusual high precision to all environmental changes, presenting a high learning rate about contextual volatility that jeopardizes the learning of probabilistic distributions of variant stimuli^30^. Other findings outlined the typical integration of overall statistics of environments, but the rate of updating internal priors may be slower^31^. These perspectives are not mutually exclusive but may be triggered by different contexts and experimental conditions. For instance, environments that requested pre-existing social priors resulted most frequently in favor of strong sensorial influences or no evidence of imbalance, rather than prior influences^32^.

In the present study, affective speech prosody acts as task-irrelevant distractor while children with low (typically developed, TD) and high autistic traits (diagnosed with ASD) were involved in both intermediate and high visual attentional loads tasks, and in neutral images viewing (low load). Multiple-Object Tracking (MOT) paradigms were chosen because of their high reliability to capture effects of experimental manipulations on attentional demand, and because performance of MOT tasks was previously well-characterized on both TD and autistic populations^33,34^. This work was designed to explore affective prosody perception as distractor under visual attentional load modulations. This is the first study evidencing neuronal spectral time-course (ERSP) in the balance between bottom-up and top-down systems during emotional prosody processing and correlating it with autistic traits. We expect children with high-autistic traits to (a) present disturbed and effortful emotional integration evidenced by lower theta and high-beta ERS, higher gamma ERS, lower alpha and low-beta ERD, and (b) have emotional processing less affected by concurrent attentional load variations than children with low-autistic traits as well as lower MOT tracking capacities because of lower ability to segregate relevant content from noisy information.

## Materials and methods

This study has been registered at BioMed Central under the following number: ISRCTN18117434 (last access on January, 31st, 2023)^35^.

### Participants

Sixty-two children were recruited for the experiment (mean: 10-year-old; standard deviation: 1). One parent or legal guardian of each child completed the Spanish version of the Autism Spectrum Rating Scale (ASRS)^36^ to estimate the magnitude of autistic traits. The full-length ASRS was used, which consists of 71 items to assess autistic behaviors divided into the following scales: (1) Social/Communication (SC), (2) Unusual Behaviors (UB), (3) Self-Regulation (SR), (4) Total Score (TOT; SC, UB, SR), (5) DSM-5 criteria, (6) Peer Socialization (PS), (7) Adult Socialization (AS), (8) Social/Emotional Reciprocity (SER), (9) Atypical Language (AL), (10) Stereotypy (ST), (11) Behavioral Rigidity (BR), (12) Sensory Sensitivity (SS), and (13) Attention (AT). TOT is an equally weighted composite of SC, UB and SR. A 4-level scale is presented to specify how often a particular behavior is observed (0 = Never, 1 = Rarely, 2 = Occasionally 3 = Frequently, 4 = Very frequently). Standardized scores (T-scores) are based upon the relationship between a child’s raw score and scores for children of the same age included into the normative sample representing the typical population. T-scores equal to or greater than 60 mean that higher ratings were attributed than what is observed in the normative sample. Higher T-scores indicate higher symptoms severity.

**Figure 1:**
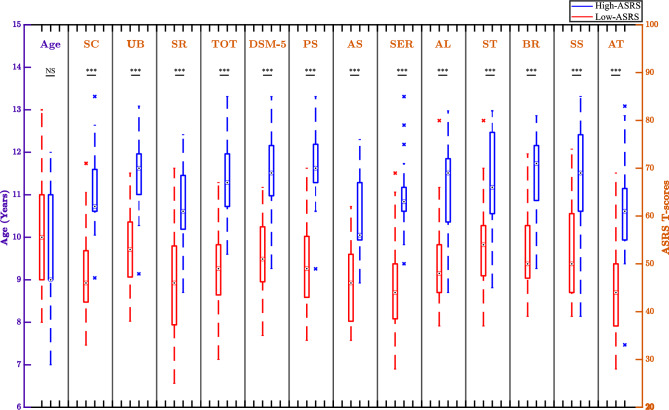
Participants’ demographic characteristics as regards age and autistic symptomatology. ASRS, Autism Spectrum Rating Scales; SC, social/communication; UB, unusual behaviors; SR, self-regulation; TOT, total score, DSM-5 criteria; PS, peer Socialization; AS, adult socialization; SER, social/emotional reciprocity; AL, atypical language; ST, stereotypy; BR, behavioral rigidity; SS, sensory sensitivity; AT, attention. A repeated-measure two-way ANOVA was conducted to assess the effects of scales and groups (high- and low-ASRS) on T-scores (groups: F(1,30) = 94.50, p-value = 8.804e−11, η^2^_G_ = 0.46). Mauchly’s test of sphericity was used to evaluate homogeneity of variances and co-variances. Because of violation of sphericity (p-value < 0.05), a Greenhouse–Geisser correction was performed (scales: F_GGe_(1,30) = 0.29, p-value = 2.374e−09, Groups:Scales: F_GGe_(1,30) = 0.36, p-value = 0.27). Also, normality of residuals was assessed and confirmed with Shapiro–Wilk test (W = 0.995, p-value = 0.170). Post-hoc analyses were performed by Tukey’s procedure. A sensitivity analysis was performed to assess the influence of outliers (i.e., values out of 1.5 times the interquartile range) on statistical significance. The ANOVA was conducted without outliers and was robust to their exclusion. For the “Age” variable, the statistical result was obtained using an independent sample t-test between high-ASRS and low-ASRS. Normality was assessed and confirmed with Shapiro–Wilk test (W_Low-ASRS_ = 0.934, p-value_Low-ASRS_ = 0.07; W_High-ASRS_ = 0.882, p-value_High-ASRS_ = 0.06). No skewness nor presence of outliers was outlined. Homogeneity of variances was confirmed (F = 0.657, p-value = 0.25). “***” p < 0.001, NS: Non significance.

Half of children (n = 31; mean: 10-year-old; standard deviation: 1; 20 girls) were part of the “low-ASRS” group. Detailed demographic characteristics are presented in Fig. 1. Parents or guardians reported that they had no history of language, cognitive, hearing, psychiatric, or psychological pathology and were not under medication affecting central or peripheral nervous system at the time of the study.

The other half (n = 31, mean: 10-year-old; standard deviation: 1; 2 girls) was part of the “high-ASRS” group**.** Parents or guardians of those children reported the presence of autistic disorders with severity level 1 for social/communication and restricted, repetitive behaviors, or Asperger’s syndrome previously detected by experienced clinicians. Instruments reported were: Diagnostic and Statistical Manual of Mental Disorders, Fifth Edition^37^ (n = 2), Childhood Autism Spectrum Test^38^ (n = 1), Mexican filter for Asperger’s detection^39^ (n = 17), Ángel Rivière’s autism spectrum inventory^40^ (n = 2), Autism Diagnostic Interview–Revised^41^ (n = 3), Autism Diagnostic Observation Schedule, Second Edition^42^ (n = 4), Psychoeducational Profile, Third Edition^43^ (n = 1), Gilliam Autism Rating Scales, Third Edition^44^ (n = 1). High-ASRS children were not diagnosed with any other condition and were not under medication that could have affected central or peripheral nervous systems at the time of the study. Importantly, the unbalanced representativity of genders between and within groups may have limited the generalizability of further results and interpretations.

The internal reliability of every scale as measured by Cronbach’s alpha using R software is presented in Table 1. Cronbach’s alpha ranges from 0 to 1 with the highest consistency at 1. Here, coefficients indicate that items within every scale are closely related when assessing behaviors of both high- and low-ASRS children.

**Table 1:** Cronbach’s alpha measured on every scale used to assess behaviors of high- and low-ASRS children.

Scale	Number of items	High-ASRS	Low-ASRS
SC	19	0.88	0.84
UB	24	0.90	0.86
SR	17	0.86	0.91
TOT	60	0.93	0.93
DSM-5	34	0.92	0.89
PS	9	0.80	0.70
AS	6	0.88	0.84
SER	13	0.77	0.86
AL	6	0.78	0.88
ST	5	0.79	0.58
BR	8	0.91	0.76
SS	6	0.69	0.65
AT	11	0.86	0.86

All participants were Mexican, currently living in Mexico, with Spanish as their mother-tongue, had normal or corrected-to-normal vision, and received academic and familial Mexican educations. According to the Spanish version of the Edinburgh Handedness Inventory^45^, nine children from the low-ASRS group were ambidextrous and one was left-handed while the others were right-handed (mean laterality quotient = 62.5; standard deviation = 42.9), one child from the high-ASRS group was ambidextrous and two were left-handed while the others were right-handed (mean laterality quotient = 80.6; standard deviation = 45).

Sample size was previously determined by an a priori power analysis for cluster-based permutation model with four predictors: (1) means, (2) standard deviation (a significant cluster in the theta band was simulated over the post-stimulus period), (3) minimum correlation between paired samples of 0.5, and (4) power of 0.9 to detect emotion, condition, and interaction effects for a within-subject design^46^. This analysis revealed that a sample size of 21 participants was required to obtain 90% power. Sample size estimation for group, condition, and interaction effects for a within-subject design was assessed using G*Power^47^ for 0.5 effect size, minimum correlation between paired samples of 0.5 and 0.9 power on a repeated-measure ANOVA. The statistical level was set at p < 0.05. The analysis revealed that at least 20 participants were required to reach 90% power. Following the same methodology, post-hoc sensitivity analyses were conducted to evaluate the robustness of statistical analyses and assess the extent to which a greater sample size (n = 31) affected the power of statistical results. For the cluster-based permutation model, this analysis revealed that a sample size of 23 participants was required to obtain 90% power, and 97% power was reached with 31 subjects in each group. For the repeated-measure ANOVA model, this analysis revealed that a sample size of 19 participants was required to obtain 90% power, and 94% power was reached with 31 subjects in each group.

### Ethics statement

This study was conducted according to the guidelines of the Declaration of Helsinki and approved on November 30th, 2021, by the Ethics Committee of the School of Medicine of Tecnologico de Monterrey (register number within the National Committee of Bioethics CONBIOETICA 19 CEI 011-2016-10-17) under the following number: P000409-autismoEEG2020-CEIC-CR004. A written informed consent was obtained from all the children and one of their legal guardians.

### Stimuli and procedure

The auditory stimuli were taken from the Mexican Emotional Speech Database (MESD)^48–50^, available in Mendeley Data at http://doi.org/10.17632/cy34mh68j9.5. For this inventory, 24 single-word utterances per emotion (anger, disgust, fear, happiness, neutral, and sadness) were intoned by a Mexican female speaker. Participants were seated comfortably in an armchair in front of a computer screen while listening to the auditory stimuli and their EEG activity was recorded. OpenVibe (1.3.0) was used to design the auditory paradigm and to record EEG signals. During EEG recordings, children were exposed to 3 tasks displayed on the computer screen. The task sequence was randomly distributed across participants. Two of them were unique-target Multiple-Object Tracking (MOT) tasks and the other consisted of viewing neutral images from the International Affective Picture System^51^ (IAPS condition).

**Figure 2:**
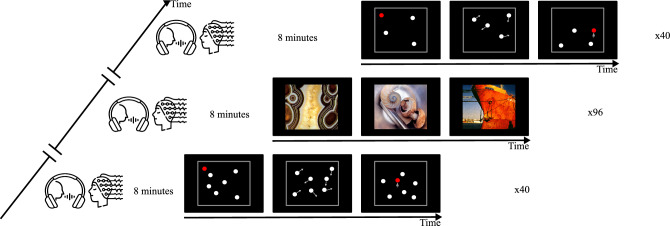
Experimental set-up. Participants were presented with two MOT and one image-viewing tasks while listening to emotional utterances and EEG recordings. The order of tasks was randomly distributed across participants.

Utterances were displayed at 70 dBA via the Shure SRH1840 audio headset that has a flat frequency response to accurately reproduce the input audio signal. Stimuli were presented consecutively with a 3.11 s stimulus-onset asynchrony for an eight-minute task. The sequence was randomly distributed and differed for every participant. This procedure was repeated for each of the 3 visual stimuli conditions, for an entire 24-min task (3 times eight minutes). Children were allowed to take a break as long as they required between each condition (each 8-min task) to focus for the following task. Procedures are illustrated in Fig. 2.

MOT trials started with the appearance of an empty grey square frame (20 × 20 degrees of visual angle) on a black background for 500 ms. White discs of 1.3° of visual angle diameter were further displayed with random positions inside the frame (either 4 or 8 for one task or the other). In both tasks, one disc (the target for visual tracking) immediately started flashing in red four times with 200 ms intervals to finally remaining red during 400 ms and becoming white again. Then, all discs started moving for 8 s with an 8° of visual angle (deg/s) speed following random straight paths inside the frame. Whenever a disc touched an edge, it was reflected with an angle equal to the incidence one. After motion, all discs remained motionless positioned at their latest location and the mouse appeared on the screen. Children could then use the wireless mouse located next to the computer on their dominant side (right for right-handers, left for left-handers) to select the target disc that they were able to track. In case tracking was lost during movement, it was instructed to guess and mark a random disc. Participants did not receive feedback to avoid inducing emotions. The next trial immediately started after selecting one disc. Children were instructed to remain with their hand on the mouse all-task to reduce muscle and movement artefacts on EEG signals. Every child completed the same 40 trials in the same order to avoid unwanted variance from trial order and maximizing differences arising from individual heterogeneity.

Instructions were explained both verbally and in writing, and participants were told to ask all questions needed before starting the session. Before starting the first MOT task, 3 practice trials with 6 discs and 1 target were presented to familiarize the child with the task. At the beginning of the experiment, participants were asked to relax for 60 s, and get prepared to focus on the task.

PsychoPy3 (3.2.4) was used to generate all tasks (MOT and IAPS conditions). MOT tasks were generated by editing the open-source code shared by Meyerhoff and Papenmeier^52^.

Images for the IAPS condition were selected according to ratings for valence, arousal and dominance provided by the Technical Report A-8^51^. Neutral pictures were those which valence, arousal, and dominance ratings were between 4 and 6 (both excluded) according to ratings from all subjects. Considering ratings from children was not convenient for this study due to the small number of pictures rated by this sample. Pictures finally included are described in Table 2. They were presented on a black background with size 20 × 20 degrees of visual angle. Each image stayed on screen for 5 s before the following picture appeared. As well as MOT tasks, IAPS condition lasted 8 min during which emotional utterances were listened to.

**Table 2:** Neutral pictures included in IAPS condition.

Number	Description	Number	Description	Number	Description	Number	Description
3005.2	Gold	7054	Glass	7461	FrenchFries	7830	Agate
5395	Boat	7077	Stove	7476	Ramen	8211	Sailboat
5455	Cockpit	7092	Scale	7487	Pastry	8325	RaceCars
5535	Stilllife	7188	AbstractArt	7496	Street	9422	Battleship
5661	Cave	7211	Clock	7504	Stairs	9468	Grafitti
5900	Desert	7247	AbstractArt	7560	Freeway	9472	Bridge
6900	Aircraft	7248	AbstractArt	7620	Jet		
6910	Bomber	7365	Meat	7632	Airplane		
7013	Lightbulb	7402	Pastry	7820	Agate		

For all three tasks, participants were instructed not to pay attention to the auditory stimuli but focus on the visual task. That is, auditory stimuli were task-irrelevant distractors.

### Tracking capacity (TC)

The tracking capacity (*m*) index (i.e., corrected for guessing) was computed from MOT behavioral results. Whenever the number of targets successfully tracked was smaller than the number of targets, the child had to guess the remainder. Because only one disc on the screen was the target, the rate of correct guess was 1/number of discs (*d*). Thus, the proportion of correctly identified targets (*p*) was defined by Eq. (1)^53^:

1 $$p= n/d (m/n+d/2)$$

where *n/d* is the simplified equation of the number of targets (*n*) divided by the number of discs (*d*), and *m* is the effective number of targets tracked.

Rearrangement of Eq. (1) yields Eq. (2):

2 $$m=n(dp-d/2)$$

Therefore, *m* is in range [$-d/2 \,\, d/2$]. To enable comparisons between MOT tasks, we rescaled TC indexes in range [− 1 1] using Eq. (3):

3 $${m}^{^{\prime}}= -1+\left(2(m-\mathrm{min}(m))/\mathrm{max}\left(m\right)-\mathrm{min}(m) \right)$$

where:

- min(*m*) = − 2 when *d* = 4,
- min(*m*) =  − 4 when *d* = 8,
- max(*m*) = 2 when *d* = 4, and
- max(*m*) = 4 when *d* = 8. *m’* is the rescaled *m.*

### EEG recording and processing

**Figure 3:**
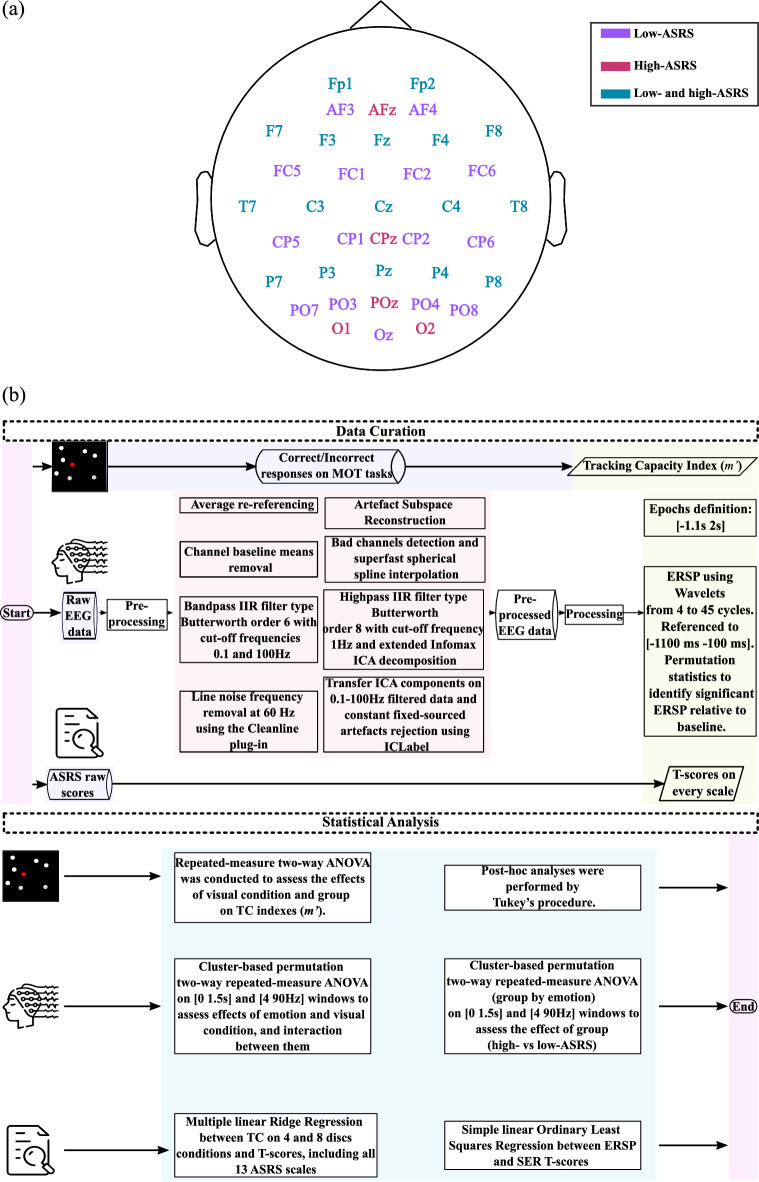
EEG data acquisition and analysis. (**a**) Sensors’ topography. (**b**) Data curation and statistical analysis.

For technical reasons, different EEG set-ups were used for low- and high-ASRS children. A topoplot is presented in Fig. 3a that describes sensors for both systems. For the low-ASRS group, continuous EEG data were acquired at 256 Hz sampling rate from a 32-channel EEG amplifier system (gUSBamp, gTec) with Ag/AgCl scalp electrodes placed according to the modified expanded 10–20 system on the GAMMAcap3 headset. Electrodes included: Fp1, Fp2, AF3, AF4, F7, F3, Fz, F4, F8, FC5, FC1, FC2, FC6, T7, C3, Cz, C4, T8, CP5, CP1, CP2, CP6, P7, P3, Pz, P4, P8, PO7, PO3, PO4, PO8, Oz. During online recording, AFz was used as ground, and data was referenced to the left earlobe. Electrode impedance was kept below 5 kΩ.

For the high-ASRS group, the mBrain Train Smarting mobi semi-dry setup with 24 active electrodes was used to record continuous EEG (sampling rate: 500 Hz). Channels were placed according to the modified expanded 10–20 system. Electrodes included: Fp1, Fp2, F3, F4, C3, C4, P3, P4, O1, O2, F7, F8, T7, T8, P7, P8, Fz, Cz, Pz, A1, A2, AFz, CPz, POz. During online recordings, the Common Mode Sense (reference) electrode was located at FCz and the Driven Right Leg electrode (ground) was placed at Fpz. Electrode impedance was kept below 10 kΩ.

EEGLab toolbox version 2021.0 from Matlab was used to pre-process and process the data. Figure 3b summarizes EEG data pre-processing, processing, and statistical analysis. Data were offline average re-referenced. Then, channels baseline means were removed and a bandpass IIR filter type Butterworth order 6 with cut-off frequencies 0.1–100 Hz was applied. Line noise frequency at 60 Hz was removed using the Cleanline plug-in. Then, high variance spontaneous artefacts were removed by the Artefact Subspace Reconstruction algorithm^54^. On average 22.5 out of 24 trials remained after artifacts rejection. Bad channels were depicted as (1) having a flatline longer than 5 s, or (2) presenting more line noise relative to signal than 4 standard deviations based on the total channel population, or (3) showing joint log probability falling more than 5 standard deviations from the mean of the probability density function of the whole channel population. Once bad channels were detected and removed, they were interpolated by the superfast spherical spline interpolation method (m = 4, n = 7). On average, 3.4 out of 32 channels were interpolated for the low-ASRS group, and 1.1 out of 24 channels were interpolated for the high-ASRS group. Interpolating more channels on data from low-ASRS did not weaken subsequent analyses as no hypothesis was raised regarding the topography. The signal was further decomposed into Independent Components (extended Infomax ICA) and the ICLabel plug-in was used to reject constant fixed-sourced artefacts (components that were in the brain category with less than 70% confidence were rejected). On average 2 components were rejected for the low-ASRS group, and 1.54 components for the high-ASRS group.

EEG data were epoched over a 3.11 s time window (1.11 s pre-, 2 s post-stimulus). Sample rate was equalized to 256 Hz for both groups before data processing. Event-Related Spectral Perturbations (ERSP) were computed in the 4–90 Hz frequency range using Morlet wavelets expanding from 4 cycles at lowest frequency to 45 at highest. ERSP data were referenced to a 1000 ms silent baseline extracted from the [− 1100 − 100] interval with a surrogate distribution generated from 200 randomly sampled time points within this baseline window. Permutation statistics were computed to identify significant ERSP relative to baseline (p < 0.05). ERSP were time-locked to auditory stimuli (and not to visual inputs) to reflect emotion perception rather than additional processes such as attentional reallocation between visual and auditory stimuli, or precision-weighting between sensorial inputs and priors, and updating processes after expectation-prediction errors integration.

### Statistical analysis

#### Tracking capacity

Statistical analysis was performed with R software (R Foundation for Statistical Computing, Vienna, Austria). The level of significance was set at p < 0.05. A repeated-measure two-way ANOVA was conducted to assess the effects of visual conditions and groups (high- and low-ASRS) on TC indexes (*m’*). Homogeneity of variances and co-variances (as tested by Mauchly’s test of sphericity) was not violated as the model had only two within-subject levels. Also, normality of residuals was assessed and confirmed with Shapiro–Wilk test (W = 0.984, p-value = 0.142). Post-hoc analyses were performed by Tukey’s procedure. A sensitivity analysis was performed to assess the influence of outliers on statistical significance. The ANOVA was conducted without outliers and was robust to their exclusion.

#### ERSP

A non-parametric cluster-based permutation repeated-measure two-way ANOVA was used to assess the effects of both categorical variables (i.e., emotion and visual condition) on ERSP, and the interaction between them. This analysis was implemented to assess whether both variables acted concomitantly on the ERSP.

Cluster-based permutation tests were implemented to explore emotion and visual conditions effects within both low- and high-ASRS groups, and group effect within visual conditions, using Fieldtrip Toolbox. Conditions were compared at every sample (channel × frequency × time) by means of univariate repeated-measures ANOVA on a [0–1.5 s] and [4–90 Hz] windows. Samples were clustered based on temporal, spatial and spectral adjacency whose F-value was larger than a critical threshold (p < 0.05). Clusters were formed by two or more neighboring sensors. Then, cluster-level statistics were computed by the sum of F-values within every cluster. The maximum cluster-level statistic was taken. To evaluate cluster-based statistics, clustering was combined with non-parametric permutation analysis, with 1000 random shuffling across conditions under the null hypothesis of data exchangeability. For each permutation, cluster-based statistics were calculated, and a distribution was built. The proportion of random partitions that resulted in a larger test statistic than the one that was actually observed was the p-value that was used to assess the effect. The Monte-Carlo estimate was used. When comparisons from ANOVA were significant, post-hoc analyses were performed by means of non-parametric cluster-based permutation dependent samples T-tests. P-values for significance were adjusted for two-sided tests (p < 0.025) and corrected for multiple comparisons with the Bonferroni method.

Finally, a repeated-measure two-way ANOVA was implemented to assess group effect on ERSP. When comparisons from ANOVA were significant, post-hoc analyses were performed by means of non-parametric cluster-based permutation dependent samples T-tests to compare high- and low-ASRS individuals on ERSP for each emotion. Only sensors in common between both groups were considered (see Fig. 3a). p-values for significance were adjusted for two-sided tests (p < 0.025) and corrected for multiple comparisons with the Bonferroni method.

In case of non-significant results, a Bayesian repeated-measure ANOVA was conducted to quantify the evidence in favor of the null hypothesis (absence of effect) and confirm the lack of effect rather than the insensitivity of the data to substantiate the alternative or the null hypothesis. Bayes factors were computed using JASP software^55^. The BF_01_ factor was reported, that indicates the likelihood of the data to occur under the null hypothesis as compared to the alternative hypothesis^56^. Particularly, BF_01_ equal to B means that the data are B times more likely to exist under the null hypothesis. When BF_01_ is 1, data do not favor any of the hypotheses, and are ambiguous. BF_01_ is in range [0 ∞[, where values under 1 indicate data in favor of the alternative hypothesis, BF_01_ greater than 3 or less than 1/3 indicate “moderate” evidence, greater than 10 or less than 1/10 are “strong evidence”, and greater than 30 or less than 1/30 are “very strong”. Any BF between 1/3 and 3 is considered “anecdotal”.

#### Linear effects of autistic symptomatology on TC and ERSP

Finally, the relationship between behavioral or neuronal patterns, and daily-life autistic symptomatology was tested.

A multiple linear regression analysis was implemented to assess linear effects of autistic behaviors on TC using R software. First, Variance Inflation Factors (VIF) were calculated for each of the independent variables. High multicollinearity was found for most of the variables (VIF ≥ 10). Therefore, a linear Ridge regression model minimizing Eq. (4) was implemented to regularize regression parameters and provide acceptable estimators correcting the variance inflation of variables affected by multicollinearity. Generalized Cross-Validation was used to choose the optimum value for Ridge penalty parameter.

4 $$\sum_{i=1}^{N}{({y}_{i}-{\widehat{y}}_{i})}^{2}+ \lambda \sum_{k=1}^{K}{a}_{k}^{2}$$

where *i* are observations, N is the total number of observations, *k* are independent variables, *K* the total number of them, *a* are the partial slope coefficients, and λ is the penalty parameter. This analysis was performed to assess the scales of daily-life behaviors that were related to behavioral patterns measured within the experimental set-up.

The Linear Ordinary Least Squares (OLS) method was applied to fit a regression model between ERSP computed on EEG data while processing each emotional prosody and T-scores on SER. As ERSP were directly linked to emotional processing, SER was the only scale that allowed a coherent interpretation of the results. Average values over significant clusters for emotion effect were computed and regressed. Independence, normality, and homogeneity of residuals were assessed by Durbin-Watson, Shapiro–Wilk, and Breusch-Pagan tests, respectively. P-values were corrected from multiple comparisons with the Bonferroni procedure.

Outliers were detected by bivariate boxplots which are two-dimensional analogues of traditional boxplots for univariate data. Outliers were excluded from regression models. On average 2.4 values were detected as outliers for the multiple linear regression model, and 2.8 values were outliers for the OLS model. Sensitivity analyses were performed to assess the effect of outliers on regression outputs by conducting regression models without removing outliers. The same methodology was followed. Results from sensitivity analyses are available in Supplementary Information (Table S1 for the multiple linear regression analysis, and Table S2 for the OLS model). Ridge and OLS regression models were significantly affected by the presence of isolated outliers and failed to provide efficient estimates, inducing a bias towards those values that are non-representative of the general sample.

## Results

### Tracking capacity is shaped by attention load and autistic traits

Both attentional load conditions (MOT 4-disc and MOT 8-disc) and groups (high- and low-ASRS) acted significantly on TC (groups: F(1,30) = 28.28, p-value = 9.49e−06, η^2^_G_ = 0.19; load: F(1,30) = 12.97, p-value = 1.13e−03, η^2^_G_ = 0.042), and the interaction between both variables was significant (F(1,30) = 6.14, p-value = 1.90e-02, η^2^_G_ = 0.02). Low-ASRS children presented higher TC during the 4-disc condition (z = − 3.560, p-value = 0.002, mean 4-disc = 0.79, mean 8-disc = 0.65). No difference between both attentional load conditions was observed for high-ASRS (z = − 0.497, p-value = 0.960, mean 4-disc = 0.39, mean 8-disc = 0.33). High-ASRS children presented poorer TC than low-ASRS during both 4-dics and 8-disc conditions (4-disc: z = 6.292, p-value < 0.001, 8-disc: z = 3.229, p-value = 0.0069).

### Four-disc MOT condition is optimal for emotion processing by low-ASRS

**Figure 4:**
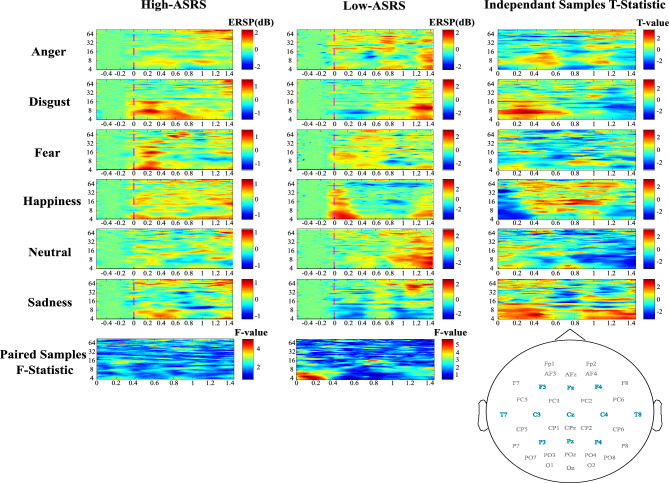
Event-Related Spectral Perturbations (ERSP; Frequency oscillations: Hz as regards time: s) and statistical comparisons during MOT 4 discs. ASRS: Autism Spectrum Rating Scales. Data were averaged over blue sensors as represented on the topoplot that are common between both groups, and where emotion effect occurred for low-ASRS.

Emotion recognition was mostly highlighted during the intermediate attentional load (MOT 4-disc condition) for low-ASRS children. Figure 4 illustrates ERSPs on every emotion for high- and low-ASRS with F- and T-statistics for emotion and group comparisons, and Table 3 details statistical outputs.

**Table 3:** Statistical outputs for emotion and group effects during MOT 4 discs.

Group	Effect	Cluster	Sum (statistic)	p-Value	Time (s)	Frequency (Hz)	Topography
Low-ASRS	Emotion	1	59,919	0.001	[0 0.5]	[4 11]	F3, Fz, F4, FC5, FC1, FC2, FC6, T7, C3, Cz, C4, T8, CP5, CP1, CP2, CP6, P3, Pz, P4
		2	25,748	0.003	[0.8 1.4]		
		3	11,352	0.026	[0.6 1]	[12 18]	
		4	8822.8	0.04	[0 0.7]	[29 34]	
	Disgust–Sadness	1	9670.5	0.019	[0.8 1.4]	[6 19]	
	Fear–Happiness	1	14,910	0.015	[0.2 1.3]	[11 18]	
	Fear–Sadness	1	11,137	0.016	[0.9 1.4]	[4 6]	
		2	8615	0.021	[0 0.4]	[4 10]	
	Happiness–Neutral	1	21,914	0.005	[0 0.3]	[4 7]	
	Happiness–Sadness	1	24,133	0.011	[0 0.4]	[4 8]	
	Neutral–Sadness	1	23,566	0.001	[0.7 1.4]	[4 11]	
	Anger–Happiness	1	− 15,847	0.008	[0 0.4]	[4 7]	
	Anger–Neutral	1	− 8479.9	0.004	[0.7 1.2]	[8 11]	
	Disgust–Fear	1	− 15,087	0.011	[0.1 0.5]	[4 9]	
	Disgust–Happiness	1	− 26,047	0.01	[0 0.3]	[4 7]	
	Disgust–Neutral	1	− 3707.2	0.02	[0 0.4]	[6–8]	
High-ASRS vs Low- ASRS	Group	1	10,526	0.028	[0 1.3]	[4 13]	
		2	8543.2	0.032	[0.4 1.4]	[12 22]	
	Anger	1	− 1930.8	0.024	[0.5 1.3]	[4 7]	C4
	Disgust	1	3050.6	0.016	[0 0.6]	[4 13]	Fz
		2	2980	0.017	[0 0.6]	[4 13]	F3
		3	2808	0.022	[0 0.8]	[4 9]	C4
		1	− 4435.3	0.004	[0.4 1.4]	[4 22]	C3
	Fear	1	− 1429.1	0.06	[0 1.3]	[7 13]	Fz
	Happiness	1	2477.2	0.014	[0.2 1.1]	[9 22]	Fz
		2	− 2342.9	0.017	[0 0.4]	[4 11]	C4
	Neutral	1	− 2926.2	0.011	[0.2 1.4]	[6 12]	T8
		2	− 2137.7	0.024	[0.9 1.4]	[4 13]	Cz
	Sadness	1	2119.6	0.028	[0 0.5]	[4 11]	Cz

No cluster was found for the high-ASRS group (BF_01_ = 4.33).

IAPS and 8-disc conditions did not promote emotion recognition by either low- or high-ASRS children. Statistical outputs are presented in Table 4.

**Table 4:** Statistical outputs for emotion effect during IAPS for low-ASRS and group effects during MOT 8 discs and IAPS.

Group/condition	Effect	Cluster	Sum (statistic)	p-Value	Time (s)	Frequency (Hz)	Topography
Low-ASRS/IAPS	Emotion	1	3702.6	0.04	[0.4 0.7]	[13 16]	F3, Fz, F4, FC5, FC1, FC2, FC6, T7, C3, Cz, C4, T8, CP5, CP1, CP2, CP6, P3, Pz, P4
	Anger vs Happiness	1	6639.3	0.008	[0.3 0.7]	[13 20]	
High-ASRS vs Low ASRS/ 8-disc	Group	1	18,526	0.022	[0.6 1.2]	[4 15]	T7, C3, Cz, C4, T8
	Happiness	1	1732.9	0.03	[0.6 1.2]	[4 7]	T7
		2	1475.4	0.04	[0.6 1.2]	[4 7]	C3
High-ASRS vs Low-ASRS/ IAPS	Group	1	11,123	0.026	[0 1.4]	[4 11]	C3, Cz, C4, T8, P3, Pz, P4
		2	7413.3	0.04	[0.4 1.4]	[13 20]	
	Neutral	1	− 4338.3	0.001	[0 1.4]	[6 14]	C4
		2	− 2283.3	0.016	[0.3 1.4]	[6 10]	Cz
	Anger	1	1824.4	0.03	[0.1 0.8]	[4 7]	C3
	Disgust	1	1567.1	0.03	[0.5 1.3]	[11 20]	C3
	Fear	1	− 1856.6	0.03	[0 1.1]	[8 16]	P3
		2	− 1681.6	0.04	[0.2 1.4]	[8 13]	P3

MOT 8-disc did not trigger any significant cluster for emotion effect in the low-ASRS group (BF_01_ = 34.23). No cluster was found for emotion effect in the high-ASRS group (BF_01_ = 18.39).

Surprisingly, the attention load effect was significant for low-ASRS (sum(F) = 44,603, p-value = 0.004, time(s) = [0 3.7], frequency(Hz) = [4 7], topography: F3, Fz, F4, FC5, FC1, FC2, FC6, T7, C3, Cz, C4, T8, CP5, CP1, CP2, CP6, P3, Pz, P4) with significant comparisons only for happiness with two significant post-hoc comparisons of same topography (8-disc vs 4-disc, sum(T) = 9816, p-value = 0.01, time(s) = [0 3.6], frequency(Hz) = [4 7], IAPS vs 4-disc, sum(T) = − 21,828, p-value = 0.002, time(s) = [0 3.9], frequency(Hz) = [4 7]). No cluster was found for high-ASRS (BF_01_ = 7.48).

### Emotion and attention load act concomitantly on ERSP only in typically developed children

**Figure 5:**
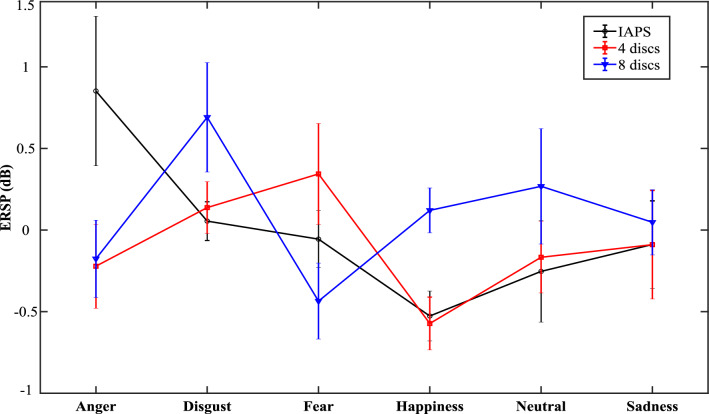
Emotion and attention load act concomitantly on Event-Related Spectral Perturbations (ERSP) for low-ASRS. Data have been averaged over topography, time, and frequency window of significance.

A significant cluster for the qualitative interaction between attentional load and emotion was highlighted for low-ASRS (sum(F) = 6619.1, p-value = 0.03, time(s) = [0.3 0.8], frequency(Hz) = [13 18], topography: F3, Fz, F4, FC5, FC1, FC2, FC6, T7, C3, Cz, C4, T8, CP5, CP1, CP2, CP6, P3, Pz, P4). This interaction is represented in Fig. 5. No cluster was found for high-ASRS (BF_01_ = 54.29).

### Tracking capacity and event-related spectral perturbations are predicted by daily-life autistic behaviors

Multiple Ridge regression outlined significant linear relation between TC during both 4-disc and 8-disc conditions and UB, TOT, DSM-5, and AL T-scores of the ASRS, revealing a progressive decrease in TC with higher autistic traits. The overall statistical output for the 4-disc model is: p-value for F-test(3.49, 47.98) = 0.09; k = 0.57; variance: 0.11; bias: 88.68, and is: p-value for F-test(2.56, 55.20) = 0.1; k = 1.09; variance: 0.12; bias: 33.59 for the 8-disc model. Estimates, their standard error, T-values, and p-values are detailed in Table 5.

**Table 5:** Multiple linear Ridge regression outputs for 4-disc and 8-disc models to predict TC.

Independent variable	Estimate		Standard error		T-Value		p-Value	
	4-disc	8-disc	4-disc	8-disc	4-disc	8-disc	4-disc	8-disc
SC	− 0.02	− 0.07	0.09	0.09	− 0.18	− 0.68	0.86	0.5
UB	− 0.13	− 0.16	0.07	0.07	− 1.93	− 2.35	**0.06 **	0.02*
SR	− 0.05	− 0.1	0.08	0.09	− 0.61	− 1.15	0.55	0.25
TOT	− 0.09	− 0.12	0.04	0.04	− 2.24	− 2.97	0.03*	0.004**
DSM-5	− 0.11	− 0.12	0.04	0.05	− 2.68	− 2.58	0.01*	0.01*
PS	− 0.05	− 0.04	0.09	0.09	− 0.51	− 0.44	0.61	0.66
AS	− 0.10	− 0.04	0.11	0.10	− 0.96	− 0.36	0.34	0.72
SER	− 0.12	− 0.10	0.09	0.09	− 1.38	− 1.16	0.17	0.25
AL	− 0.35	− 0.41	0.12	0.13	− 2.86	− 3.06	0.006**	0.003**
ST	− 0.04	− 0.11	0.12	0.12	− 0.31	− 0.91	0.76	0.37
BR	− 0.01	− 0.01	0.10	0.10	− 0.12	− 0.09	0.91	0.92
SS	− 0.07	− 0.16	0.10	0.10	− 0.63	− 1.58	0.53	0.12
AT	− 0.12	− 0.1	0.10	0.10	− 1.20	− 0.94	0.24	0.35

Significant values are in bold.

“*” p < 0.05, “**” p < 0.01.

Ordinary Least Squares simple regression between ERSP and T-scores on SER revealed significant linear relations detailed in Table 6. Particularly, higher autistic severity was linked with higher early theta synchronization, lower early alpha desynchronization, higher late low-beta synchronization, and higher late alpha desynchronization.

**Table 6:** Ordinary least squares simple regression outputs for SER T-scores to predict ERSP over significant sensor-time–frequency clusters for emotion recognition.

Dependent variable	Estimate	Standard error	T-Value	p-Value
ERSP Cluster 1—Anger	0.02	0.007	2.43	0.16
ERSP Cluster 1 Theta—Disgust	0.03	0.009	2.96	0.04*
ERSP Cluster 1—Fear	0.02	0.006	3.83	0.0024**
ERSP Cluster 1 Alpha—Fear	0.014	0.006	2.18	0.24
ERSP Cluster 1 Theta—Fear	0.03	0.01	3	0.032*
ERSP Cluster 3—Happiness	0.02	0.006	3.1	0.024*
ERSP Cluster 2—Neutral	− 0.02	0.007	− 2.87	0.048*
ERSP Cluster 2 Alpha—Neutral	− 0.02	0.008	− 2.11	0.32

Significant values are in bold.

“*” p < 0.05, “**” p < 0.01, and “***” p < 0.001; Clusters are significant ones for emotion effect during 4-disc condition detailed in Table 3.

## Discussion

The current study aimed to investigate whether emotional prosodies are differently recognized during implicit perception under concurrent attention load modulations. Task-irrelevant emotion processing was assessed by ERSP from EEG data analysis. Besides, dynamic (tracking a target over time) and selective (differentiating target from non-target) attentional abilities were evaluated by the behavioral TC index during MOT tasks. For the first time, neurological indices of implicit emotion integration, and a behavioral marker of attentional abilities were correlated with autistic traits severity.

First, in line with our hypothesis, the emotional integration by high-ASRS children was insensitive to attention load variations and higher autistic traits were correlated with lower tracking capacity, highlighting lower segregation of relevant sensorial information from noise. Second, low-ASRS children presented high prioritization for emotional perception at intermediate attention load (4-disc MOT), mild prioritization at low load (image viewing) but no emotion-related attentional bias was observed during high load (8-disc MOT). Additionally, attention load and emotion acted concomitantly on ERSP related to emotional recognition. Finally, only partially consistent with our hypothesis, higher autistic traits were associated with impaired emotion processing that was outlined by (a) early alpha ERS (disgust, sadness, fear) but a tendency to lower late ERS (disgust, neutral) or alpha ERD (fear), (b) late theta ERD for anger, lower early and late ERS for happiness and neutral, but higher early and late ERS for disgust, fear, and sadness, (c) late low-beta ERS (happiness), compared with typically developed children. Results are thereafter discussed.

Attentional load was conveyed by MOT tasks that required both motion and selective attention processes, making it difficult to distinguish between local dynamic hindrance (inaccurate evaluation of the direction of individual discs) and contextualization problems (difficulty refraining attention to noisy dots) to explain lower TC performance of children with autism in both 4- and 8-disc conditions. Nevertheless, previous works outlined typical accuracy to estimate individual motions in autistic children^57,58^ and similar MOT performance deficit at higher speeds, highlighting no impairment in dynamic attention^59^. Thus, the current results may be interpreted within a Bayesian perspective of selective attention, suggesting that autism may be characterized by a failure to down-modulate the sensorial processing of task-irrelevant information, i.e., non-target discs, or to efficiently update the perceptual statistical patterns of ongoing contexts^60,61^. In other words, higher autistic traits may show an imbalance ascribed to sensory precision relative to hidden states reliance, towards excessive weight of sensory information against a given goal^62^. In this scheme, distractor dots may be extensively processed by high-ASRS children during both 4- and 8-disc scenarios, which may explain lower performance and lack of benefit from a lower attention load conveyed by the 4-disc condition.

Consequences of inadequate contextualization of sensory information may be extended to autistic hypo- and hyper-sensitivity, unusual, stereotyped and repetitive behaviors, atypical communication and delicate social interactions^37^. Indeed, inappropriate adjustments of perceptions in proportion to hidden states driven by an over reliance on sensations may trigger both excessive and poor attentional allocation to sensory aspects of the environment. Accordingly, this atypical updating in precision between sensations and prior knowledge may lead to impairments in predicting events, as perception would be mostly driven by sensory inputs. Consequently, unexpected events may be overwhelming resulting in insistence on sameness. Besides, motor stereotypy may arise from inflexibility and failure to contextualize. Lastly, poor adjustments of precision may induce deficits in maintaining and understanding social and emotional behaviors driven by a lack of contextualization of events. Heterogeneity in precision-weighting shapes individual differences in perception since balance must be constantly updated in terms of learned expectation but become atypical with higher autistic traits. This framework may explain the significant negative relation between tracking capacity during MOT and autistic severity for UB, TOT, DSM-5, and AL observed in the current study. Nevertheless, the other dimensions of the autistic symptomatology measured by the ASRS failed to predict the experimental observations. We hypothesize that the Bayesian framework may not equally conform to every aspect of autistic behaviors, and other mechanisms may be involved.

Low-ASRS children presented optimal emotion discrimination during the 4-disc condition in contrast with IAPS and 8-disc conditions which led to poor task-irrelevant emotion perception. We suggest that attentional load induced by the 4-disc scenario triggered the depletion of top-down cognitive control that would inhibit auditory noisy information processing, resulting in higher likelihood to attentional allocation and integration of emotional content. In contrast, during IAPS condition which required lower cognitive resources, affective arousal was reduced when mediated by the suppression of attentional allocation to task-irrelevant distractors by executive systems. We found a significant interaction between the effect of attentional load and prosody on neuronal marker of emotion integration, confirming that both affective content and concomitant cognitive load modulated emotional recognition. A surprising finding was that the 8-disc scenario did not increase the likelihood of attentional allocation and recognition of task-irrelevant emotional information, although lower TC confirmed higher attentional load. Cognitive effort is known to deactivate brain structures involved in the Default Mode Network (DMN), initially identified for its functioning at rest^63^. Its greater activity was associated with internally oriented processes such as mind-wandering, and situational self- and social representations, as well as remembering past events and planning future ones. Yet, the functional activity of the DMN is also locked into external events such as engagement into social interactions^64^. Besides, parietal and frontal areas of the DMN contribute to emotion understanding and regulation based on social evaluation^65^. Additionally, frontal, temporal, and parietal brain structures that compose the DMN are involved in cognitive representational mechanisms to differentiate between discrete emotions^66^. The disruption of affective information recognition during higher attentional load may have been driven by the deactivation of DMN brain regions associated with affect processing.

Emotion recognition by TD children was characterized by differential ERSP patterns among discrete affective prosodies in early (0–0.5 s) and late (0.8–1.4 s) theta and alpha, late (0.6-1 s) low-beta, early and late (0–0.7 s) gamma bands over frontal, fronto-central, central, temporal, centro-parietal, and parietal cortices. Early attentional allocation outlined by alpha oscillations were characterized by both ERS and ERD, observing ERS during high-arousal stimuli perception (anger, fear, and happiness). Alpha-mediated inhibitory processes are involved in both selective attention allocation and affective arousal perception for which optimized neuronal processing is driven by concurrent disinhibition of relevant and inhibition of irrelevant brain systems reflected respectively by alpha ERD and ERS of local electric fields. Nevertheless, synchronization (desynchronization) may be generalized over the scalp whenever inhibited (disinhibited) regions are broader, more numerous or more decently aligned with the EEG sensors^67^. In line with previous studies^68,69^, in the current experimental context, the integration of high-arousing stimuli may mostly rely on the disengagement of irrelevant representations. Another relevant factor is the presence of a competing task, in this case the MOT scenario, which could have modulated the balance between inhibitory and disinhibitory mechanisms. Indeed, processing highly motivational affective information that acted as irrelevant distractor within attentional load could have biased early alpha oscillations towards neuronal inhibition of emotion-irrelevant mechanisms, attentional top-down executive control systems, and MOT-relevant brain areas. In contrast, later stages were characterized by a short period of alpha ERD (anger: 0.4–0.6 s, fear: 0.8–1.2 s, happiness: 0.7–1.1 s), outlining an overall disinhibition of relevant emotion-processing areas, followed by top-down inhibitory control for return to resting state at late stages (1.2–1.4 s) observed for all emotions.

As expected, low-ASRS children presented late low-beta ERD (anger: 0.4–0.6 s, fear: 0.8–1.4 s, happiness: 0.7–1.1 s, sadness-early/late: 0–1.4 s) that may depict mirror neurons activity over frontal, central, parietal, and temporal cortices engaged in social and affective cognition. Furthermore, theta ERS was outlined over frontal, temporal, and parietal cortices at early and late stages as an index of long-term memory encoding, retrieval, and associations with internal representations for emotional apprehension. Nevertheless, early, and late low-beta ERS and theta ERD (disgust: 0–0.5 s, sadness: 0–0.4 s, 0.8–1.4 s) also occurred. On the one hand, low-beta ERS may be associated with the activity of the salience network that could have been responsible for an emotional “freezing” in response to relevant stimuli^70^. On the other hand, low-beta ERS considered in association with theta ERD may have reflected cognitive inhibitory control (faster oscillations) on affective integration (slower waves), typically observed during spontaneous emotion regulation, and associated with better emotion recognition performances^71^.

This work’s novelty relies on the characterization of the autistic condition suggesting inadequate excessive early cortical inhibition (alpha ERS) while processing negative emotions (disgust, sadness, fear) that is diminished (disgust) or inverted (ERD; sadness and fear) at later stages. Besides, low-beta ERSP outlined early, late ERS and significant relation between late synchronization and higher SER autistic patterns (happiness: 0.6–1 s), indicating lesser social and emotional affective integration at higher autistic severity. Accordingly, late theta ERD (anger: 0.7–1 s), lower early (happiness: 0–0.4 s) and late (neutral: 0.9–1.4 s) ERS were observed. Surprisingly, higher early and late ERS were highlighted for disgust (0–0.8 s), sadness (0–0.5 s), and higher early (0–0.5 s) ERS was related to higher SER autistic patterns (disgust and fear). Theta oscillations are involved in shared mechanisms between memory and lexico-semantic processing^72^. To that extent, fronto-temporal ERS underlies lexical access and semantic memory retrieval, and greater activity is indicative of effortful cognitive engagement^73^. Thus, higher autistic traits may be associated with extensive recruitment of resources for lexical meaning integration under emotionally and attentionally demanding conditions.

To sum up, time–frequency analyses on neuronal integration of affective prosody outlined atypical patterns and poor emotion recognition associated with higher autistic traits. Lower tracking capacity during MOT and insensitivity of both behavioral and neuronal data to attention load manipulations may suggest aberrant precision to sensorial information. The over-focus on fine-grained inputs might be disadvantageous for affective prosody understanding, driving the attention away from contextual evaluations. For children with lower autistic traits, an optimized emotion integration was triggered by concurrent intermediate attention load that may encourage cognitive control resources depletion without inhibiting social cognition.

Some limitations must be noted. First, when searching for attention load effect on emotion processing, a significant cluster was found only for happiness perception by low-ASRS children. Although the emotion effect was present during the 4-disc condition only, this result outlines a relatively small power of attention load effect on emotion integration. This observation may explain why previous studies highlighted emotion recognition during low cognitive demand by using different visual and affective prosody stimuli^3,14^. Second, children with high- and low-autistic traits were detected by scores on the ASRS, and parents of high-ASRS children confirmed previous diagnosis established by a clinician. Nevertheless, no formal confirmation of diagnosis was assessed at the time of the study. Last, a different set-up was used to record EEG signals for low- versus high-ASRS children, which might be a source of raw data modulations. To ensure that this methodological concern was not triggering the observed differences between groups, we conducted a cluster-based permutation independent samples T-test to compare high- and low-ASRS individuals on data recorded at rest (µV/ms; pre-processed) with eyes open while viewing a white cross under a black background on a computer screen for 2 min. Clustering was based on temporal and spatial adjacency (0 or more neighboring sensors) and 1000 permutations were used. No significant cluster was highlighted over the whole spatiotemporal window. Additionally, sample rates, referencing, and frequency bandwidth were equalized between groups during pre-processing. Thus, EEG set-ups did not cause significant divergences between both groups. Finally, although more sensors were used to record data from low-ASRS children, emotion effects were observed on shared electrodes. Therefore, emotion processing could be compared between low- and high-ASRS children.

## Supplementary Information

Supplementary Information.

## Data Availability

The datasets generated from this study have been made available in Mendeley Data^74^ at http://doi.org/10.17632/spwnt8t25y.1^75^ and http://doi.org/10.17632/7zf99hxxc9.1^76^.
