# Supplementary material for: Autistic traits shape neuronal oscillations during emotion perception under attentional load modulation
Source: Sci Rep. 2023 May 20;13:8178. doi: 10.1038/s41598-023-35013-x (PMC10199905; doi:10.1038/s41598-023-35013-x)
Supplement: Supplementary file 1 — Supplementary Information. [file 41598_2023_35013_MOESM1_ESM.pdf]

Autistic traits shape neuronal oscillations during emotion perception under attentional load modulation

Mathilde Marie Duville [a], *, David I. Ibarra-Zarate [a] and Luz María Alonso-Valerdi [a]

[a] *Tecnologico de Monterrey, Escuela de Ingeniería y Ciencias, Ave. Eugenio Garza Sada 2501, Monterrey, N.L., México, 64849*

* Correspondence: a00829725@tec.mx

Table S1 presents the statistical outputs of the sensitivity analysis (with outliers) by multiple Ridge regression for T-scores to predict Tracking Capacity (m'). The overall statistical output for the 4-disc model is: P-value for F-test(3.46, 57.00)=0.04; k=0.57; variance: 0.37; bias: 89.43, and is: P-value for F-test(2.56, 58.20)=0.14; k=1.09; variance: 0.18; bias: 66.95 for the 8-disc model. Estimates, standard errors, T-values, and P-values are detailed in Table S1.

Table S1. Multiple linear Ridge regression outputs of the sensitivity analysis for 4-disc and 8-disc models to predict Tracking Capacity (TC, m'). "*" p<0.05, "**" p<0.01. Outliers were not removed from the regression models. SC: Social/Communication, UB: Unusual Behaviors, SR: Self-Regulation, TOT: Total Score, DSM-5 criteria, PS: Peer Socialization, AS: Adult Socialization, SER: Social/Emotional Reciprocity, AL: Atypical Language, ST: Stereotypy, BR: Behavioral Rigidity, SS: Sensory Sensitivity, AT: Attention.

**Independent variable**	**Estimate**		**Standard Error**		**T-Value**		**P-Value**	
	4 discs	*8 discs*	*4 discs*	*8 discs*	*4 discs*	*8 discs*	*4 discs*	*8 discs*
**SC**	-0.12	-0.11	0.17	0.12	-0.71	-0.94	0.48	0.35
**UB**	-0.22	-0.16	0.12	0.08	-1.88	-1.89	**0.07.**	**0.06 .**
**SR**	0.039	-0.10	0.15	0.11	0.25	-0.91	0.80	0.36
**TOT**	-0.13	-0.14	0.07	0.05	-1.98	-2.75	**0.053 .**	**0.008 ****
**DSM-5**	-0.33	-0.11	0.08	0.06	-4.12	-1.84	**0.0001 *****	**0.07 .**
**PS**	-0.02	-0.16	0.17	0.11	-0.12	-1.46	0.90	0.15
**AS**	0.17	0.017	0.20	0.12	0.85	0.13	0.40	0.89
**SER**	-0.21	-0.001	0.16	0.11	-1.32	-0.01	0.19	0.99
**AL**	-0.44	-0.46	0.23	0.16	-1.91	-2.85	**0.06 .**	**0.006 ****
**ST**	-0.35	-0.12	0.21	0.15	-1.71	-0.79	**0.09 .**	0.43
**BR**	-0.06	-0.01	0.19	0.12	-0.35	-0.09	0.73	0.93
**SS**	-0.11	-0.11	0.20	0.13	-0.55	-0.87	0.58	0.39
**AT**	-0.19	0.18	0.18	0.13	-1.01	-1.35	0.32	0.18

Table S2 presents the statistical outputs of the sensitivity analysis (with outliers) by Ordinary Least Squares simple regression for SER T-scores to predict Event-Related Spectral Perturbations (ERSP).

Table S2. Ordinary Least Squares simple regression outputs of the sensitivity analysis for SER T-scores to predict ERSP over significant sensor-time-frequency clusters for emotion recognition. "*" p<0.05, "**"

$p<0.01$, and "***" $p<0.001$; Clusters are significant ones for emotion effect during 4-disc condition detailed in Table 3. Outliers were not removed from the regression model.

**Dependent variable**	**Estimate**	**Standard Error**	**T-Value**	**P-Value**
**ERSP Cluster 1 - Anger**	0.004	0.009	0.51	0.61
**ERSP Cluster 1 Theta - Disgust**	0.03	0.01	2.24	**0.029 ***
**ERSP Cluster 1 - Fear**	0.01	0.01	0.90	0.38
**ERSP Cluster 1 Alpha - Fear**	0.007	0.01	0.49	0.63
**ERSP Cluster 1 Theta - Fear**	0.01	0.01	0.90	0.38
**ERSP Cluster 3 - Happiness**	0.02	0.02	1.52	0.14
**ERSP Cluster 2 - Neutral**	-0.03	0.02	-1.28	0.21
**ERSP Cluster 2 Alpha - Neutral**	-0.02	0.02	-1.14	0.26
